# Study on the Correlation between Metabolism, Insulin Sensitivity and progressive weight loss change in Type-2 Diabetes

**DOI:** 10.12669/pjms.36.7.3027

**Published:** 2020

**Authors:** De-Xian Kong, Yan-xin Xiao, Zhen-Xi Zhang, Ya-Bin Liu

**Affiliations:** 1 De-Xian Kong, Department of Endocrinology, The Fourth Affiliated Hospital of Hebei Medical University, Shijiazhuang, Hebei 050011, P.R. China; 2 Yan-xin Xiao, Department of Endocrinology, Baoding No.1 Central Hospital, Baoding, Hebei, 071000, P.R. China; 3 Zhen-Xi Zhang, Department of General Surgery, Peoples Hospital of Linxi County, Xingtai, Hebei, 054900, P.R. China; 4 Ya-Bin Liu, Department of General Surgery, The Fourth Affiliated Hospital of Hebei Medical University, Shijiazhuang, Hebei 050011, P.R. China

**Keywords:** Insulin sensitivity, Lipid metabolism, Progressive weight loss, Type 2 diabetes

## Abstract

**Objective:**

To observe the changes of lipid metabolism, blood glucose level and insulin sensitivity in patients with Type-2 diabetes after progressive weight loss of their body weight, so as to lay a theoretical foundation for diabetes treatment and education in the future.

**Methods:**

One hundred obese patients with Type-2 diabetes (BMI ≥ 25 kg/m^2^) who visited the endocrinology department of our hospital from April 2017 to April 2018 were given diabetes health education, diabetic diet, exercise and other measures to control their weight. The changes of blood glucose, blood lipid, insulin level and insulin release test before weight loss (T1), and at the time points of weight loss reached 5% (T2), 10% (T3) and 15% (T4) were recorded respectively to understand the influence of progressive weight loss on relevant indexes of patients.

**Results:**

With the decrease of body weight, the differences of TC, TG, LDL-C and HDL-C at different weight loss points were significant (p < 0.05), and the changes of fasting blood glucose in 5% and 10% weight loss were significant (p = 0.02). The 2h postprandial blood glucose showed the most significant difference when the weight loss reached 15% (p = 0.00). There was no statistical difference in the change of glycosylated hemoglobin among different weight loss points (p = 0.08). When the weight loss reached 10%, the blood insulin level was significantly lower than that before the weight loss, while the insulin level was not significantly changed when the weight loss reached 15%, but the peak of secretion was shifted forward. It is suggested that insulin sensitivity gradually increases with weight loss.

**Conclusion:**

Obese patients with Type-2 diabetes can benefit from weight loss, with abnormal blood glucose and lipid metabolism improved, insulin resistance relieved, and insulin sensitivity increased.

## INTRODUCTION

With the improvement of people’s life style and changes in their living behaviors, the incidence of Type-2 diabetes is increasing year by year, which is accompanied by the prevalence of obesity and overweight.^1^ Obese patients suffer from insulin sensitivity reduction and insulin resistance formation due to the decrease of insulin receptor density in fat, thus causing hyperinsulinemia, which plays an important role in the occurrence and development of cardiovascular and cerebrovascular related diseases and neurological diseases of patients, and weight loss may constitute an important part of comprehensive treatment for patients with Type-2 diabetes.^2^-^5^ It is recommended in the guidelines for obesity that appropriate 5 - 10% weight loss can achieve significant metabolic improvement. Compared with 10% weight loss, 5% weight loss seems to be easier and has better compliance. More importantly, the metabolic benefits can be achieved by 5% weight loss, and more benefits can be realized by further weight loss.^6^-^8^ At present, no studies have found that the results of 5% weight loss are different from those of 10% and then 15% weight loss, whether in prospective or retrospective studies.

Our objective was to observe the changes of lipid metabolism, blood glucose level and insulin sensitivity in patients with Type-2 diabetes after progressive weight loss of their body weight, so as to lay a theoretical foundation for diabetes treatment and education in the future.

## METHODS

From April 2017 to April 2018, 100 obese patients with Type-2 diabetes (BMI ≥ 25 kg/m^2^) were treated in the endocrinology department of our hospital. The inclusion criteria for cases were:

1. Patients diagnosed with Type-2 diabetes according to WHO diagnostic criteria, with HbA1c of 7 - 10%, and BMI ≥ 25 kg/m^2^.
2. Patients aged between 20 and 60.
3. Patients with no liver or renal dysfunction, no gastrointestinal diseases, no endocrine diseases such as hyperthyroidism, no stress states such as fever, infection, acute myocardial infarction, surgical treatment, and no history of heavy drinking.
4. Patients who enjoyed good mental health, with the ability to cooperate with study and willingness to receive follow-up.
5. Patients who sign the informed consent form.

### Exclusion criteria for cases:

1. Patients with major organ dysfunction.
2. Patients accompanied by other diseases that obviously affect body weight.
3. Patients with previous history of malignant tumor.
4. Pregnant and lactating women.
5. Patients who suffer from mental illness but are unable to make an announcement.
6. Patients who could not participate in this study due to other diseases.

### Ethical approval

The study was approved by the Institutional Ethics Committee of The Fourth Affiliated Hospital of Hebei Medical University (dated 31^st^ May, 2020), and written informed consent was obtained from all participants.

Among these patients, there were 43 males and 57 females, aged 20 - 60 years with an average of 40 ± 11.3 years. The fasting blood glucose level was 7.9 mmol/L to 11.4 mmol/L, with an average of 9.21 ± 1.46 mmol/L, while the 2h postprandial blood glucose level was 8.6 mmol/L to 15.3 mmol/L, with an average of 13.67 ± 2.06 mmol/L. The body mass index (BMI) was 25 - 35 kg/m^2^, with an average of 29.80 ± 3.29 kg/m^2^.

### Treatment

All patients in this group were given strict diabetes health education, diabetic diet and exercise, and the drug dosage was adjusted according to the blood glucose control. For the purpose of increasing the accuracy of the study, except for four patients (0.04%) with fasting blood glucose greater than 10 mmol/L who were given insulin to interfere with blood glucose, all patients were given oral metformin to control blood glucose due to the intervention in other links of the drug is small.^2^ After comprehensive intervention, the patients lost 5% of their body weight every three months was set as a node, respectively 5%, 10%, 15%.

### Observation indexes and methods

Fasting blood glucose, 2h postprandial blood glucose, glycosylated hemoglobin, and blood lipid were collected at four time points: before weight loss (T1), 5% weight loss (T2), 10% weight loss (T3), and 15% weight loss (T4), respectively, and measured by Beckman Coulter AU680 automatic biochemical analyzer. The insulin level was detected at four time points of T1, T2, T3 and T4 respectively, and the changes of insulin release test were carried out respectively to understand the changes of insulin release test curve and serum insulin level.

### Statistical methods

All the data were statistically analyzed by SPSS 16.0 software, and the measurement data were expressed as (± S). Repeated measurement variance analysis was used for comparison of the test data, and LSD-t test was used for pairwise comparison. P < 0.05 should be regarded as statistically significant.

## RESULTS

The total cholesterol (TC), triglyceride (TG), low density lipoprotein cholesterol (LDL-C), very low density lipoprotein cholesterol (VLDL-C), high density lipoprotein cholesterol (HDL-C), apolipoprotein A1 (Apo-A1) and apolipoprotein B100 (Apo-B100) of blood lipids at different time points is shown in Table-I. Among them, TC, TG, LDL-C, HDL-C had significant difference in different weight loss points (p<0.05), T4 node has the most significant change compared with T1. However, VLDL, ApoA1 and ApoB100 had no significant difference in different nodes (p>0.05).

**Table-I:** Changes of blood lipid indexes at different weight loss time points (n = 100, *X̄*± S).

Indexes	T1	T2	T3	T4	F	P
TC (mmol/L)*	8.47±0.99	7.83±1.10	6.67±0.76	4.41±1.30	83.81	0.00
TG (mmol/L)*	3.81±1.11	3.21±1.06	2.45±0.8	1.64±0.83	45.23	0.00
LDL-C (mmol/L)*	5.74±1.32	5.31±1.22	3.38±0.73	2.13±1.03	63.41	0.03
VLDL-C (mmol/)	2.08±0.74	2.06±0.76	2.07±0.78	2.02±0.78	0.43	0.73
HDL-C (mmol/)*	1.04±0.30	1.13±0.28	1.29±1.13	1.49±0.23	8.46	0.02
ApoA1 (g/L)	1.47±1.14	1.42±1.23	1.45±1.32	1.45±1.25	0.45	0.76
ApoB100 (g/l)	1.10±0.18	1.16±0.15	1.09±0.17	1.20±0.36	1.18	0.33

* p<0.05

### Blood glucose

The changes of fasting blood glucose, 2h postprandial blood glucose and glycosylated hemoglobin after weight loss are shown in Table-II. The results show that fasting blood glucose has significant difference between 5% and 10% of weight loss (p = 0.02), while there is no significant difference between 10% and 15% of weight loss (p = 0.141). 2h postprandial blood glucose had no significant difference at 5% weight loss compared with that before weight loss (p = 0.09), but had statistic difference at 10% weight loss compared with that before weight loss (p = 0.02). The difference was most obvious at 15% weight loss (p = 0.00). In terms of the changes of glycosylated hemoglobin in different weight loss points, T1 to T3 time point showed a decreasing trend, but with no statistical difference (p = 0.08),the changes of T4 and T3 had statistical significance (p = 0.01) and had statistical significance (p = 0.01) compared with that before weight loss.

**Table-II:** Changes of blood lipid indexes at different weight loss time points (n = 100, *X̄*± S).

Indexes	T1	T2	T3	T4	F	P
Fasting blood glucose mmol/L	9.21±1.46	8.66±1.23	6.57±1.23	5.97±0.63	47.06	0.00
2 hours postprandial blood glucose mmol/L	13.67±2.06	13.22±1.84	12.82±1.87	9.80±1.57	24.90	0.01
Glycosylated hemoglobin mmol/L	8.07±0.95	7.82±0.87	7.79±0.92	5.87±1.01	26.28	0.01

### Determination of insulin level and insulin release test

### Insulin level:

**Fig.1:**
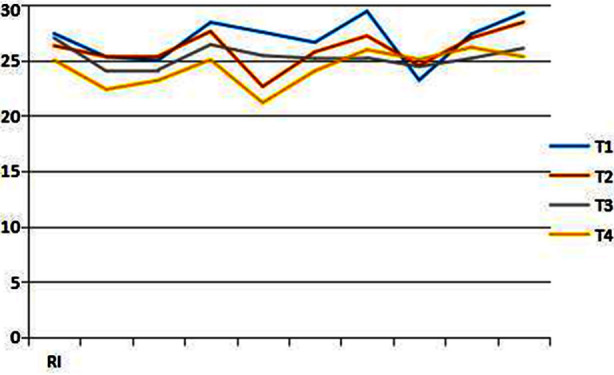
Change curve of insulin level at different weight loss time points, RI = insulin.

Insulin radioimmunoassay (RIA) was used to determine the early morning fasting blood of patients with different weight loss nodes. The measured values of insulin at different nodes were respectively: T1 26.96 ± 1.98 uu/ml, T2 26.02 ± 1.68 uu/ml, T3 25.31 ± 0.97 uu/ml, T4 24.34 ± 1.63 uu/ml. (Fig.1) Comparative analysis of insulin changes at different weight loss time points is shown in Table-III. The fasting insulin level decreased significantly when the weight loss reached 10% (p = 0.00) compared with that before the weight loss, but the insulin level did not change significantly when the weight reached 15% (T3, T4, P = 0.08) compared with that the weight loss reached 10%.

**Table-III:** Comparative analysis of insulin levels at different weight loss points.

Weight loss points	T1 T2	T1 T3	T1 T4	T2 T3	T2 T4	T3 T4
t	1.77	3.48	3.98	1.46	4.85	1.98
p	0.11	0.00	0.00	0.18	0.00	0.08

### Determination of insulin release experiment:

**Fig.2:**
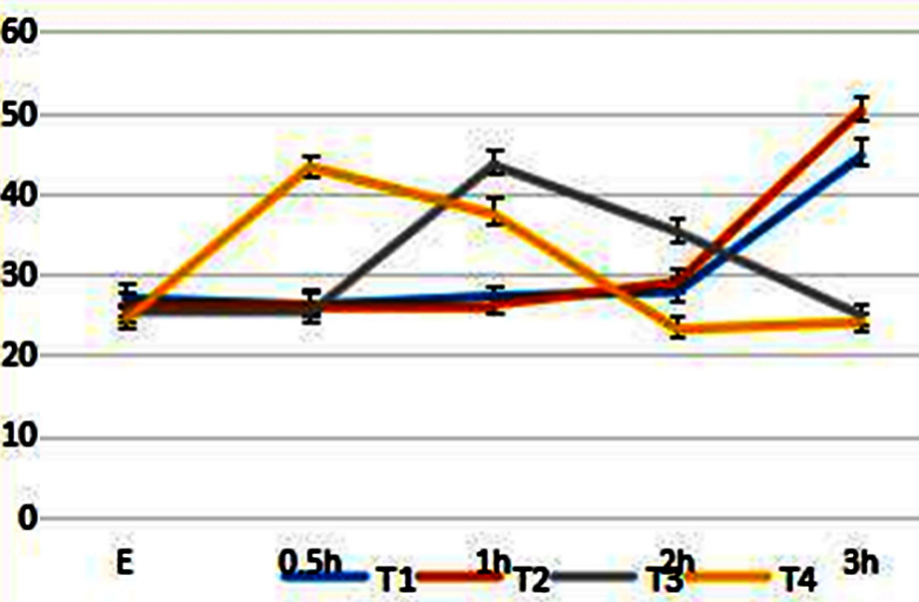
Trend chart of insulin release experiment at different weight loss points, E: fasting.

The fasting insulin was determined by RIA method in all patients in the morning, and then 75 g of anhydrous glucose was taken orally. The blood insulin level was determined at 0.5 hour, one hour, two hour and three hour after orally taking glucose. The curve of insulin release experiment showed that before weight loss and weight loss reached 5%, the peak value of insulin level increased two hours after orally taking glucose and reached two times of the basic level at about 3 hours. With the continuous weight loss, the peak value of insulin shifted forward and the rising range increased. The overall value was still higher than the normal value, but it was significantly improved compared with that before weight loss, suggesting that insulin sensitivity gradually recovered. The change trend of insulin release experiment at different weight loss time points is shown in Fig.2.

## DISCUSSION

Abnormal lipid metabolism is common in obese patients with Type-2 diabetes.^9^ Abnormal lipid metabolism is a risk factor for cardiovascular and cerebrovascular diseases such as hypertension and atherosclerosis. Study shows that TG, TC and LDL are independent risk factors for cardiovascular diseases, while HDL is a protective factor.^10^ Elevated LDL is a major risk factor for coronary heart disease.^11^ A great deal of evidences show that lipid regulation, especially LDL-C reduction, can significantly reduce the incidence of cardiovascular events.^12^ The results show that even if the obese population is not accompanied by any metabolic abnormalities, the risk of Type-2 diabetes in the future is still higher than that of normal population.^13^,^14^ Different from risk factors such as advanced age and genetics, obesity can be intervened. The results of a Swedish obesity program study show that the cumulative incidence of Type-2 diabetes in non-diabetic patients with obesity has decreased by 78% in 15 years via weight loss measures.^15^ Therefore, obesity should be identified and intervened as early as possible.

The clinical trial on diabetes relief by Faison et al. found that nearly two-thirds (64%) of the patients who lost more than 10 kg of weight got remission after two years by the implementation of weight management plan.^16^ Obesity is an important cause of insulin resistance and a major risk factor in the occurrence and development of Type-2 diabetes. Therefore, weight management is of great significance for the prevention and control of diabetes. The results of this study suggest that: with the continuous weight loss, the levels of TC, TG, HDL-C and LDL-C change obviously compared with those before weight loss, especially when the weight loss reaches 15%. It is manifested by the gradual reduction of risk factor indexes such as TC, TG, LDL-C. But HDL-C, a protective index, gradually increased. Some indexes have not yet fallen to the normal range due to the weight loss time is still short. If the weight loss is carried out for about two years as reported in the literature, various indexes of blood lipid may be further improved.

Obese patients are usually accompanied by insulin resistance, so the basal insulin secretion value of these patients is higher than that of normal people.^17^ The fasting insulin value of patients in this group before weight loss is of 26.96 ± 1.98 uu/ml (normal reference value of RIA method is of 5 - 20 uu/ml). It was confirmed that there was insulin resistance in this group of patients. Hyperinsulinemia is caused by insulin resistance or insulin insensitivity.^18^ Hyperinsulinemic blood syndrome is the common pathogenesis basis of coronary heart disease, hypertension, hyperlipidemia, Type-2 diabetes, obesity, cerebral stroke, etc., which is the most harmful to cardiovascular disease.^19^ It is currently believed that obesity can induce insulin resistance and increase the difficulty of treatment of Type-2 diabetes.^20^ By the regulation of lipid metabolism and glucose metabolism after losing weight, the response sensitivity of tissue to insulin is enhanced, and the fasting blood glucose and serum insulin level are decreased. This study confirms that the fasting insulin level decreases significantly when the weight loss reaches 10% compared with that before the weight loss (p = 0.00), but the insulin level do not change significantly when the weight reaches 15% (T3, T4, p = 0.08) compared with that the weight loss reaches 10%. It may be caused by time factors according to the analysis. However, the curve of insulin release experiment showed that before weight loss and weight loss reached 5%, the peak value of insulin level increased two hours after orally taking glucose and reached two times of the basic level at about three hours. With the continuous weight loss, the peak value of insulin shifted forward and the rising range increased. The overall value was still higher than the normal value, but it was significantly improved compared with that before weight loss, suggesting that insulin sensitivity gradually recovered.

In this study, the levels of blood lipid, blood glucose and insulin in patients with different weight loss ratios suggested that the changes of various indexes were significant when weight loss reached 15%. This study provides a theoretical basis for the management of Type-2 diabetes mellitus patients with obesity.

### Limitations of the study

(1) More data may be obtained by stratified design according to body mass index; (2) the study had a small sample size, short follow-up period and no more detailed subgroup comparison. Our findings were still needed to be further confirmed by more in-depth studies in the future.

## CONCLUSION

Obese patients with Type-2 diabetes can benefit from weight loss, with abnormal blood glucose and lipid metabolism improved, insulin resistance relieved, and insulin sensitivity increased. However, long-term and large-scale patients are still needed to further study whether patients can benefit from long-term low-weight conditions or whether further weight loss will bring better results to patients.

### Authors’ Contributions:

**DXK and** **YBL** designed this study and prepared this manuscript, and are responsible and accountable for the accuracy or integrity of the work.

**ZXZ** collected and analyzed clinical data.

**YXX** significantly revised this manuscript.
